# The prognostic value and response to immunotherapy of immunogenic cell death-associated genes in breast cancer

**DOI:** 10.3389/fonc.2023.1047973

**Published:** 2023-02-09

**Authors:** Rongling Zhao, Wenkang Wang, Limin Pan, Xuefeng Lv, Yi He, Wenping Lian, Yajie Ma, Xinyu Zhang, Ruijing Yu, Shuai Zhao, Xiaona Guo, Tao Huang, Mengle Peng

**Affiliations:** ^1^ Department of Clinical Laboratory, Henan No.3 Provincial People’s Hospital, Zhengzhou, Henan, China; ^2^ Department of Breast Surgery, The First Affiliated Hospital of Zhengzhou University, Zhengzhou, Henan, China; ^3^ Department of Breast Surgery, Zhengzhou University People’s Hospital, Henan Provincial People’s Hospital, Zhengzhou, Henan, China; ^4^ Department of Clinical Laboratory, The Third Affiliated Hospital of Zhengzhou University, Zhengzhou, Henan, China; ^5^ Department of Mini-Invasive Spinal Surgery, Henan No.3 Provincial People’s Hospital, Zhengzhou, China; ^6^ Department of Medical Affair, Henan No.3 Provincial People’s Hospital, Zhengzhou, Henan, China; ^7^ Medical School, Huanghe Science and Technology University, Zhengzhou, Henan, China

**Keywords:** immunogenic cell death, breast cancer, prognosis, genes, immunotherapy

## Abstract

Breast cancer (BRCA) remains the most prevalent cancer worldwide and the tumor microenvironment (TME) has been discovered to exert a wide influence on the overall survival and therapeutic response. Numerous lines of evidence reported that the effects of immunotherapy of BRCA were manipulated by TME. Immunogenic cell death (ICD) is a form of regulated cell death (RCD) that is capable of fueling adaptive immune responses and aberrant expression of ICD-related genes (ICDRGs) can govern the TME system by emitting danger signals or damage-associated molecular patterns (DAMPs). In the current study, we obtained 34 key ICDRGs in BRCA. Subsequently, using the transcriptome data of BRCA from the TCGA database, we constructed a risk signature based on 6 vital ICDRGs, which had a good performance in predicting the overall survival of BRCA patients. We also examined the efficacy of our risk signature in the validation dataset (GSE20711) in the GEO database and it performed excellently. According to the risk model, patients with BRCA were divided into high-risk and low-risk groups. Also, the unique immune characteristics and TME between the two subgroups and 10 promising small molecule drugs targeting BRCA patients with different ICDRGs risk have been investigated. The low-risk group had good immunity indicated by T cell infiltration and high immune checkpoint expression. Moreover, the BRCA samples could be divided into three immune subtypes according to immune response severity (ISA, ISB, and ISC). ISA and ISB predominated in the low-risk group and patients in the low-risk group exhibited a more vigorous immune response. In conclusion, we developed an ICDRGs-based risk signature that can predict the prognosis of BRCA patients and offer a novel therapeutic strategy for immunotherapy, which would be of great significance in the BRCA clinical setting.

## 1 Introduction

Breast cancer (BRCA) is one of the most common cancers, accounting for approximately 30% of female cancers, with a mortality-to-incidence ratio of 15% (1). BRCA is a biologically and clinically heterogeneous disease that can be divided into 3 recognized subtypes (hormone receptor-positive, ERBB2-positive, and triple-negative BRCA) based on the presence or absence of distinct proteins (2). Patients with different histotypes of BRCA generally have varying aetiologies and profiles, and present diverse responses to treatment and prognosis (3, 4). As for the molecular level, the most frequent pathogenic genome alternations in BRCA tend to be HER2 activation and BRCA1/2 mutations (5). Moreover, the next-generation sequencing in BRCA is largely depended on gene panels and PALB2, CHEK2, and TP53 have been identified as the key genes in BRCA development (6). Mono-allelic PALB2 germline alterations contribute to a 53% elevated risk of BRCA (7). These data emphasize the core role of genetic mutations in BRCA development and progression.

Despite the great breakthrough in cancer treatment strategies, effective clinical management for patients with metastatic BRCA remains a challenge. The standard treatment protocol includes target therapy, for example, CDK4 and CDK6 inhibitors, PI3K inhibitors, and anti-PD-L1/PD-1 immunotherapies depending on tumor subtype and molecular environment (8). The variety of treatment approaches reflects the complex nature of BRCA molecular subtypes. However, reliable prognostic markers are still lacking, hindering the improvement and individualization of therapeutic interventions (9, 10). Increasingly, calls have been made for a novel signature to prognosis prediction, with a focus on accurately identifying the overall survival and on guiding management and treatment strategies.

As a unique form of regulated cell death (RCD), immunogenic cell death (ICD), provoked by specific infectious pathogens, chemotherapeutics agents, radiation therapy, photodynamic therapy, and physicochemical therapy, is sufficient to stimulate an adaptive immune response by emitting danger signals or damage-associated molecular patterns (DAMPs) in an immunocompetent setting, in particular when it derives from cancer cells (11–13). Mechanistically, ICD induction is associated with the exposure or release of DAMPs, which operate as natural adjuvants, interacting with pattern recognition receptors (PRRs) to generate an ideal condition for the initiation of antigen-specific immune responses (14–16). The clinical response of anticancer chemotherapy application has shown that tumor-specific immune responses can determine the efficacy of anticancer therapies with conventional cytotoxic drugs. This implied that dying tumor cells in BRCA patients proceed as a vaccine that was sufficient to induce tumor-specific immune responses, thus governing or even eradicating residual cancer cells (17, 18). Indeed, the antitumor effects of extensively employed cancer treatment strategies, including conventional chemotherapy, radiotherapy, and more selective targeted therapy, are partly attributed to the induction of ICD (19).

ICD has been confirmed to serve as a novel treatment strategy by directly attacking cancer cells or induce antitumor immune responses in a broad range of solid tumors. The vaccine-like functions of ICD, indicated by transforming a “cold” tumor microenvironment to become an immunogenic, “hot” tumor microenvironment, enhances the efficacy of immunotherapy (20). The studies on ICD in BRCA are still limited. As a result, we were attempting to explore the function of ICD in BRCA by investigating ICD-related genes (ICDRGs). The mechanisms that determine ICD susceptibility to tumor cells have been researched extensively over the past several decades. However, studies on ICDRGs in BRCA are still scarce. In addition, it remains unclear whether ICDRGs were associated with BRCA prognosis. This study was designed to construct an ICDRGs risk signature in BRCA and identify its correlation with tumor immunity and immunotherapeutic response in BRCA patients. Our findings may provide clues for significant judgment and decision-making concerning BRCA treatment.

## 2 Materials and methods

### 2.1 Data collection

Captured from the recent literature, 34 ICDRGs were tailored to our study (21). FPKM transcriptome data of BRCA were sourced at the UCSC Xena database (http://xena.ucsc.edu/) from the TCGA database (https://portal.gdc.cancer.gov/). Integrating with clinical survival information, we annotated the available data, which is suitable for our further analysis. The RNA-seq data set of BRCA (GSE20711; https://www.ncbi.nlm.nih.gov/geo/query/acc.cgi?acc=GSE20711) was also retrieved from the GEO database for verification. Then, the protein-protein interaction analysis (PPI) of ICDRGs was investigated with the String database (https://string-db.org/). All the statistic analyses were performed by using the R software (version 4.0.1). For data that did not meet with the normally distribution pattern, non-parametric test was used to test the differences between different groups.

### 2.2 ICDRGs expression profile and survival analysis in BRCA

The mRNA expression was calculated employing the “limma” package in R to map the expression profile of ICDRGs in BRCA. In order to obtain differentially expressed ICDRGs between BRCA and normal samples, the P<0.05 and |fold change| >2 were considered as the criteria. Univariate cox analysis was used to obtain prognostic differentially expressed ICDRGs. Kaplan-Meier survival curve was applied for the survival analysis. Log-rank test was employed to evaluate pronounced differences in survival rates between distinct groups.

### 2.3 Construction and validation of ICD-related risk signature

Patients with BRCA from the TCGA database were randomly classified into training set and test set according to 8:2 using the “caret” package in R. We also used GSE20711 as an external dataset for verification. Univariate cox analysis was then performed to dissect prognosis-associated ICDRGs. With the purpose of further narrowing down the ICDRGs, the least absolute shrinkage and selection operator (LASSO) regression was employed in the training set. Then, an ICD-related risk signature that was capable of predicting BRCA survival was ultimately constructed in the training set by exploiting the “glmnet” R package. The prognostic risk score formula was as follows:


$$riskScore=\sum_{i=1}^{n}Coefi\;*\;\;xi$$

wherein, n, Coefi, and xi represent the number, coefficient, and corresponding expression data of prognostic ICDRGs, respectively. Patients were divided into two subgroups (ICDRGs high and low groups) based on the median risk score. Meanwhile, prognostic value of the risk model was validated in the test set, all TCGA cohort and an external verification set (GSE20711).

Next, receiver operating characteristic (ROC) curves were exploited to assess the accuracy of the risk model in predicting the overall survival of BRCA patients. Univariate and multifactor cox regression were used to determine independent risk factors. In addition, the Chi-square test was used to analyze the association between clinicopathological characteristics and ICDRGs risk groups, and the Wilcox test, together with the Krusil test was taken to analyze the risk score variations between different pathological groups.

### 2.4 The accuracy evaluation of ICD-related risk signature

Combined with the risk model and clinicopathologic characteristics, a clinical prediction column line graph (nomogram) was constructed to predict 1-, 3-, and 5-year survival of BRCA patients using the “rms” R package. Also, calibration curves and ROC curves were used to assess the predictive accuracy.

### 2.5 Drug sensitivity analysis and gene set enrichment analysis

The “pRRophetic” package was utilized to evaluate variations in the drug sensitivity between the ICDRGs high and low groups. Alternatively, the “Camp” database was used for identifying novel small molecule compounds with anti-tumor effects in the ICDRGs high or low groups. GSEA was conducted to illustrate the enrichment landscape between the ICDRGs high and low cohorts using the “clusterProfiler” package in R.

### 2.6 Characterization of the immune microenvironment in two ICDRG subgroups

We then investigated the MCPcounter, the signature scores (calculated as geometric mean) of seven immune features, and the differential expression of six immune checkpoints to unveil the immune characteristics of patients in two ICDRGs subgroups. The results could be visualized in the heatmap.

### 2.7 Prediction of response to immunotherapy

TIDE is a computational method that is capable of predicting immunotherapy response through modeling two major mechanisms of tumor immune evasion: induction of T-cell dysfunction with high cytotoxic T lymphocytes (CTL) levels and inhibition of T-cell infiltration with low CTL levels (22). Then, the TIDE score was employed to predict the immunotherapy response and cancer stem cell characters in high and low‐risk groups.

### 2.8 Immune clustering

Based on the results of MCPcounter, the BRCA patients in the TCGA cohort were stratified into distinct immune subtypes using the sum of squares of deviations. At the same time, the immune subtypes were validated in the verification set (GSE20711). Subsequently, the results of MCPcounter, the signature scores of seven immune features, as well as the differential expression of six immune checkpoints were displayed in the heatmap. The difference among the immune subgroups was also analyzed. Finally, the TIDE score was calculated to identify the immunotherapy response and tumor stemness between different immune subtypes.

## 3 Results

### 3.1 PPI network analysis of ICDRGs

**Figure 1:**
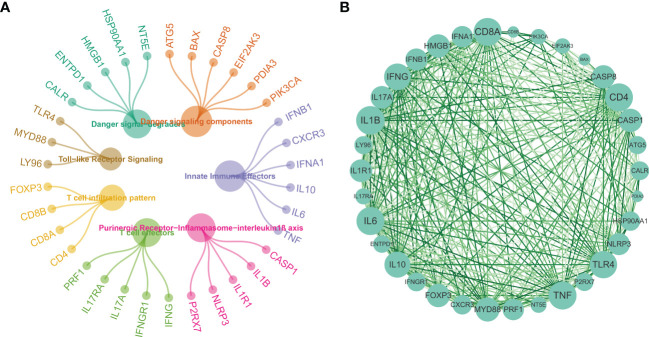
** (A, B)** Protein-protein interactions among the ICDRGs.

34 ICDRGs obtained from the recent literature were enrolled in our study for further analysis. STRING database was then proceeded to delineate the intrinsic connections among these ICDRGs by conducting the PPI network analysis. 34 ICDRGs were categorized into 7 functional subtypes, including Danger signal-degraders, Danger signaling components, Innate Immune Effectors, Purinergic Receptor-Inflammasome-interleukin1β axis, Toll-like Receptor Signaling, T cell infiltration pattern, and T cell effectors (**Figure 1A**). Furthermore, CD4, CD8A, IL6, IL1B, TNF and TLR4 exhibited the most significant interactions with other ICDRGs (**Figure 1B**).

### 3.2 Survival analysis of ICDRGs in BRCA

**Figure 2:**
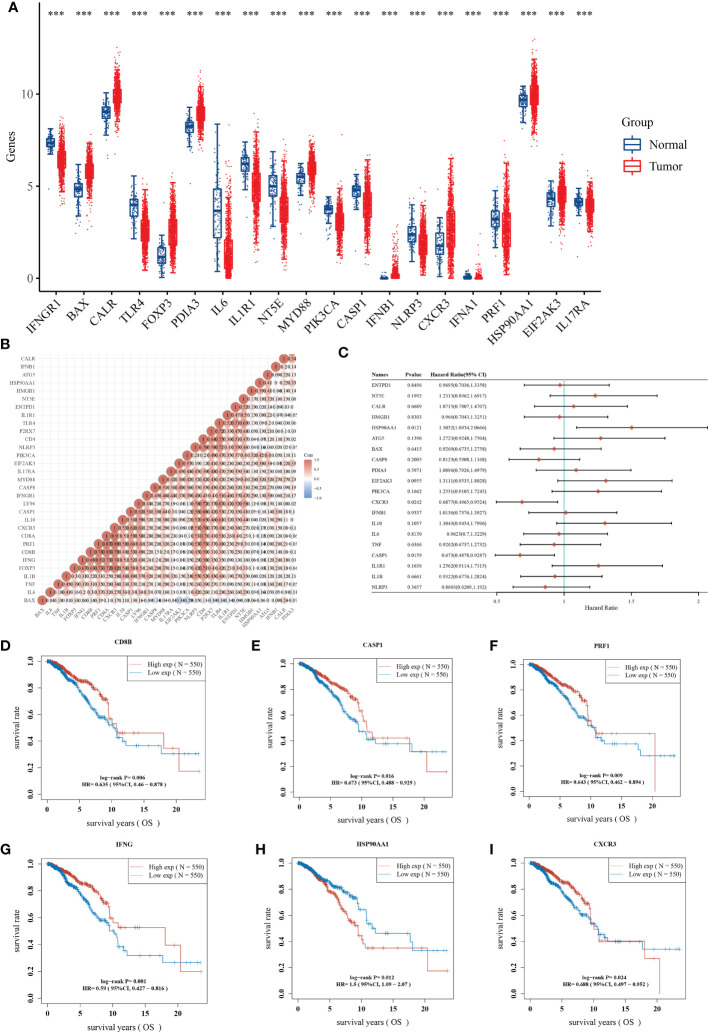
Identification of prognostic ICDRGs. **(A)** The expression profile of ICDRGs between the normal and tumor groups. **(B)** Correlation analysis of the relationship among ICDRGs. **(C)** Univariate Cox regression analysis showed the correlation between ICDRGs and BRCA prognosis. **(D-I)** Survival analysis of several ICDRGs in BRCA. **P<0.01; ***P<0.005; ****P<0.001.

We further disclosed the expression landscape of 34 ICDRGs in BRCA samples. 20 of 34 ICDRGs were differentially expressed between BRCA and normal samples, among which BAX1, CALR, PDIA3, and HSP90AA1 were overexpressed, while IFNGR1, TLR4, IL-6, IL1R1, NT5E, and PIK3CA were lowly expressed in tumor compared to normal samples (**Figure 2A**). These findings indicated the ICDRGs played a double role in BRCA. In addition, there was a positive correlation between almost each ICDRG and the others (**Figure 2B**).

Before performing univariate Cox regression analysis, we verified that cox was consistent with the hazard proportionality assumption (**Supplementary Table 1**). And univariate Cox regression analysis suggested that some differentially expressed ICDRGs (e.g., HAP90AA1, CXCR3, and CASP1) were significantly associated with the prognosis of BRCA patients (**Figure 2C**). Among them, HAP90AA1 was considered as a risk gene, while CXCR3, and CASP1 were considered as protective genes. Moreover, CD8B, CASP1, PRF1, IFNG, HAP90AA1, and CXCR3 displayed a great impact on the overall survival of BRCA patients (**Figures 2D-I**). Intriguingly, patients with high expression of HAP90AA1 generally had an unfavorable prognosis, suggesting HAP90AA1 may be an attractive candidate for predicting the survival status of BRCA (**Figure 2H**).

### 3.3 Construction of the risk signature based on ICDRGs

**Figure 3:**
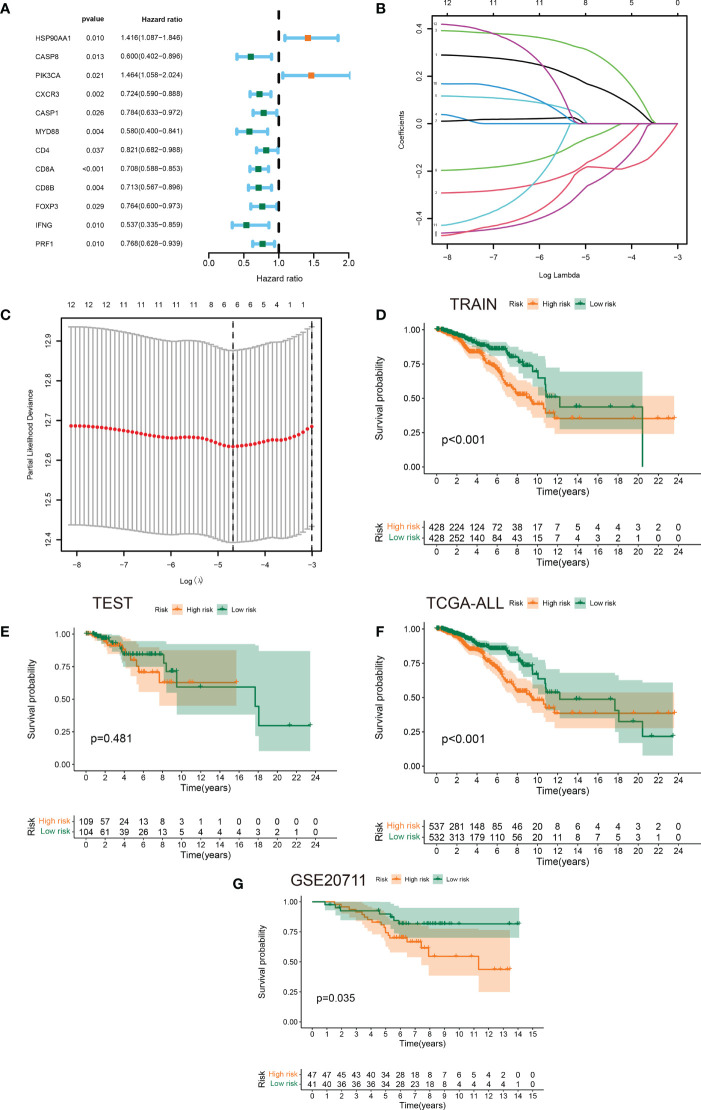
Identification of prognostic ICDRGs to establish a risk signature. **(A)** Forest plots of the results of the univariate Cox regression analysis of 12 prognostic ICDRGs in BRCA. **(B, C)** LASSO Cox regression analysis manifested that 6 out of the 10 ICDRGs were good candidates for constructing the prognostic signature. **(D-G)** Kaplan-Meier curves of patients in the high- and low-risk groups based on the 6 ICDRGs signature in the training set, test set, all TCGA cohort, and GSE20711.

Having performed a univariate cox regression analysis, 12 prognostic ICDRGs were identified (**Figure 3A**). LASSO Cox regression analysis was used to taper the ICDRGs and 6 key ICDRGs were finally figured out for the subsequent establishment of the risk model (**Figure 3B**). In addition, the partial likelihood deviance curve was plotted versus log(λ) (**Figure 3C**). The risk signature was drawn premised on the algorithm below: riskScore = HSP90AA1*0.148408207 + CASP8*(-0.136139881) + PIK3CA*(0.281022938) + MYD88*(-0.233379899) + CD8A*(-0.184609669) + CD8B*(-0.042169179). As a result, a risk signature including 6 key ICDRGs was formulated in the training set. Patients with BRCA were divided into low-risk and high-risk groups according to the median value of risk scores.The survival analysis showed that BRCA patients in the high-risk group had worse overall survival than those in the low-risk group in the training set and all TCGA cohort (**Figures 3D, F**). The test dataset also showed a similar trend, though no significant value was found, which was likely attributed to the limited samples (**Figure 3E**). In addition, another independent cohort (GSE20711) from the GEO database was used to successfully verified these results (**Figure 3G**).

### 3.4 Risk signature validation and clinical characterization analysis

**Figure 4:**
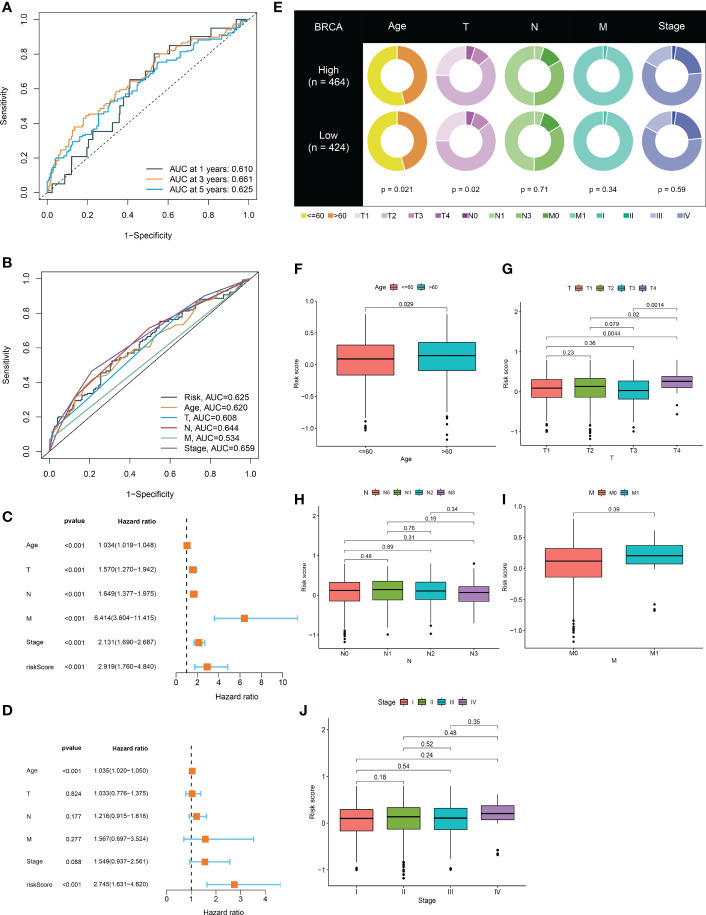
The risk model verification and its relationship with clinical characteristics. **(A)** ROC curve to evaluate 1, 3, and 5-year survival prediction efficiency of the risk model in the TCGA cohort. **(B)** ROC curve to assess prediction efficiency of the risk model, age, T stage, N stage, M stage, and pathological stage. **(C, D)** Univariate and multivariate Cox analysis showed that the risk model could be an independent prognostic indicator for BRCA patients (P<0.001). **(E)** Differences of clinical features in the high-risk and low-risk groups. **(F-J)** Clinicopathological characteristics evaluation by the risk score.

In the BRCA TCGA cohort, the ROC curves of the risk model at 1-, 3-, and 5 years demonstrated the good power of risk signature in predicting BRCA survival (AUC values were all more than 0.6) (**Figure 4A**). Additionally, in consideration of the heterogeneity of BRCA, prognostic and treatment options could be affected by clinicopathological features (23, 24). With this in mind, the ROC curves for the risk signature, age, T stage, N stage, M stage, and pathological stage were mapped in **Figure 4B**. Notably, followed by the pathological stage and N stage, the risk signature had a relatively higher AUC value, further confirming its better performance across prediction tasks (**Figure 4B**). Both the univariate Cox regression analysis and multivariate Cox regression analysis exhibited the great potential of the risk model in distinguishing the survival status of BRCA (**Figures 4C, D**). Thus, the presence of the risk signature based on ICDRGs emerged as an independent predictor of BRCA. Also, it could be viewed as a competitive candidate for the development of novel clinically valuable prognostic biomarkers. As for other clinical characteristics, age and T stage exhibited a significant difference between the high-risk and low-risk groups (**Figure 4E**). Further investigation showed that it was the existence of distinct risk scores among samples stratified by age that mattered (**Figure 4F**). Moreover, the risk score was higher for T4 and also varied for all other T stages (**Figures 4G–J**).

### 3.5 Establishment and verification of predictive nomogram

**Figure 5:**
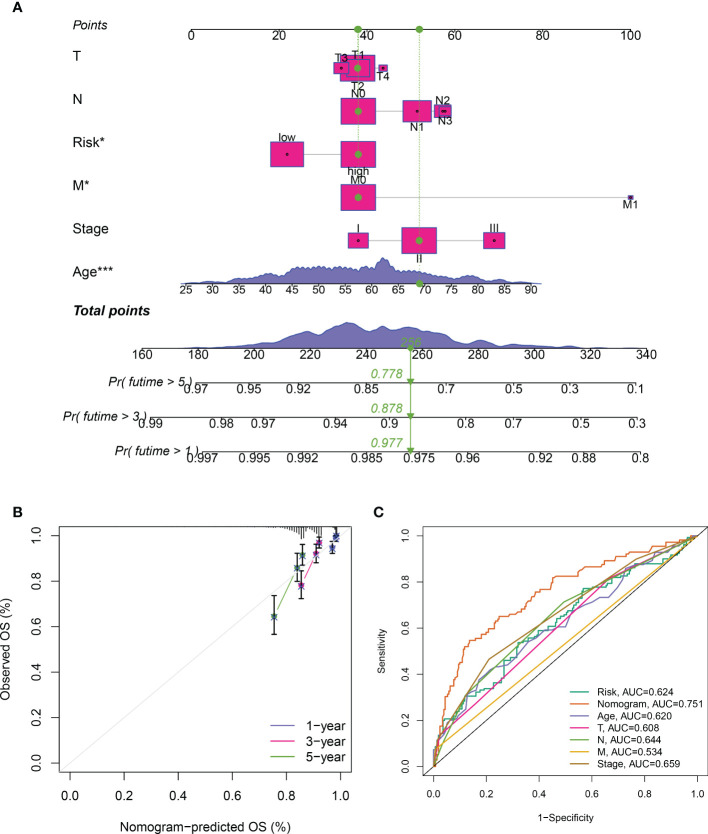
Construction of the predictive nomogram. **(A)** Predictive nomogram of the risk signature and clinicopathological characteristics. **(B)** Calibration curve of the nomogram for 1/3/5-year survival rates. **(C)** Time-dependent ROC curves for overall survival prediction of ICDRGs risk model and clinicopathological features.

To further verify the predictive efficacy, we then generated a predictive nomogram, which included the risk model, age, sex, T stage, N stage, M stage and pathological stage, to predict the 1, 3, 5-years over survival rate of BRCA patients (**Figure 5A**). Calibration plots and ROC curves presented excellent predictive accuracy of the nomogram, with the AUC value of 0.751 (**Figures 5B, C**).

### 3.6 Drug susceptibility assays

**Figure 6:**
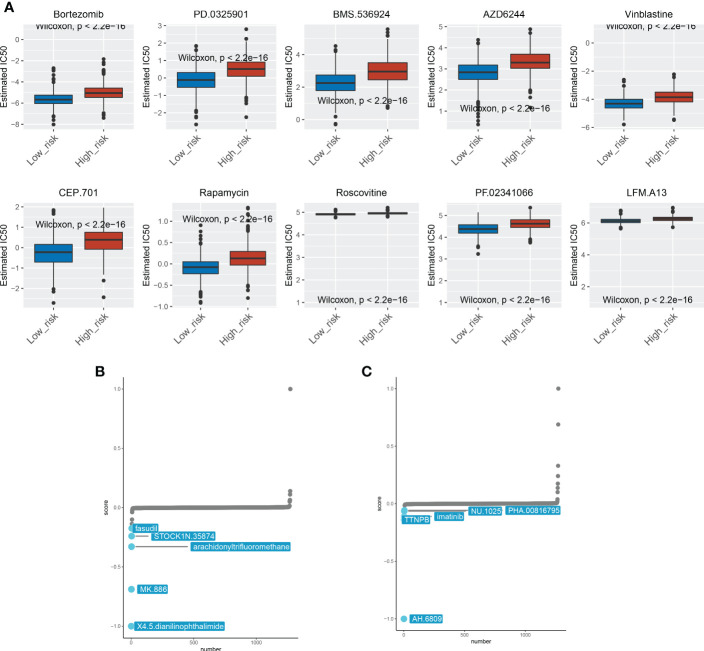
Drug sensitivity analysis between the two subgroups. **(A)** The 10 chemotherapeutic agents showed different sensitivities for patients in the high-risk and low-risk groups. **(B)** Chemotherapeutic agents sensitive to high-risk patients. **(C)** Chemotherapeutic agents sensitive to low-risk patients.

Despite improvements in cancer management and treatment, effective clinical therapies for cancer remain challenging today, primarily due to the presence of chemoresistance, which renders a deliberate obstacle to cancer care (25). Expanding literature reported that a significant proportion of patients with metastatic and local BRCA suffered from primary or acquired resistance to therapies and ultimately succumbed to disease (26, 27). Therefore, it is of great importance to illustrate the role of ICDRGs in the drug resistance of BRCA. We found that multiple chemotherapy drugs exhibited different sensitivities to high and low‐risk groups. The top 10 most paramount drugs (Bortezomib, PD.0325901, BMS.536924, AZD6244, Vinblastine, CEP.701, Rapamycin, Roscovitine, PF.02341066, and LFM.A13) manifested higher sensitivity to BRCA patients with low-risk score, and could be considered as chemotherapy agents for low-risk BRCA patients (**Figure 6A**). The Camp was used for further exploring the efficacy of small-molecule inhibitors during BRCA treatment. Patients in the high-risk group were susceptible to fasudil, TOCK1N.35874, arachidonyltrifluoromethane, MK.886, and X4.5.dianilinophthalimide, while patients in the low-risk group were impressionable to TTNPB, imatinib, NU.1025, PHA.00816795, and AH.6809 (**Figures 6B, C**). These findings may provide clues for more comprehensive clinical medication.

### 3.7 GSEA analysis

**Figure 7:**
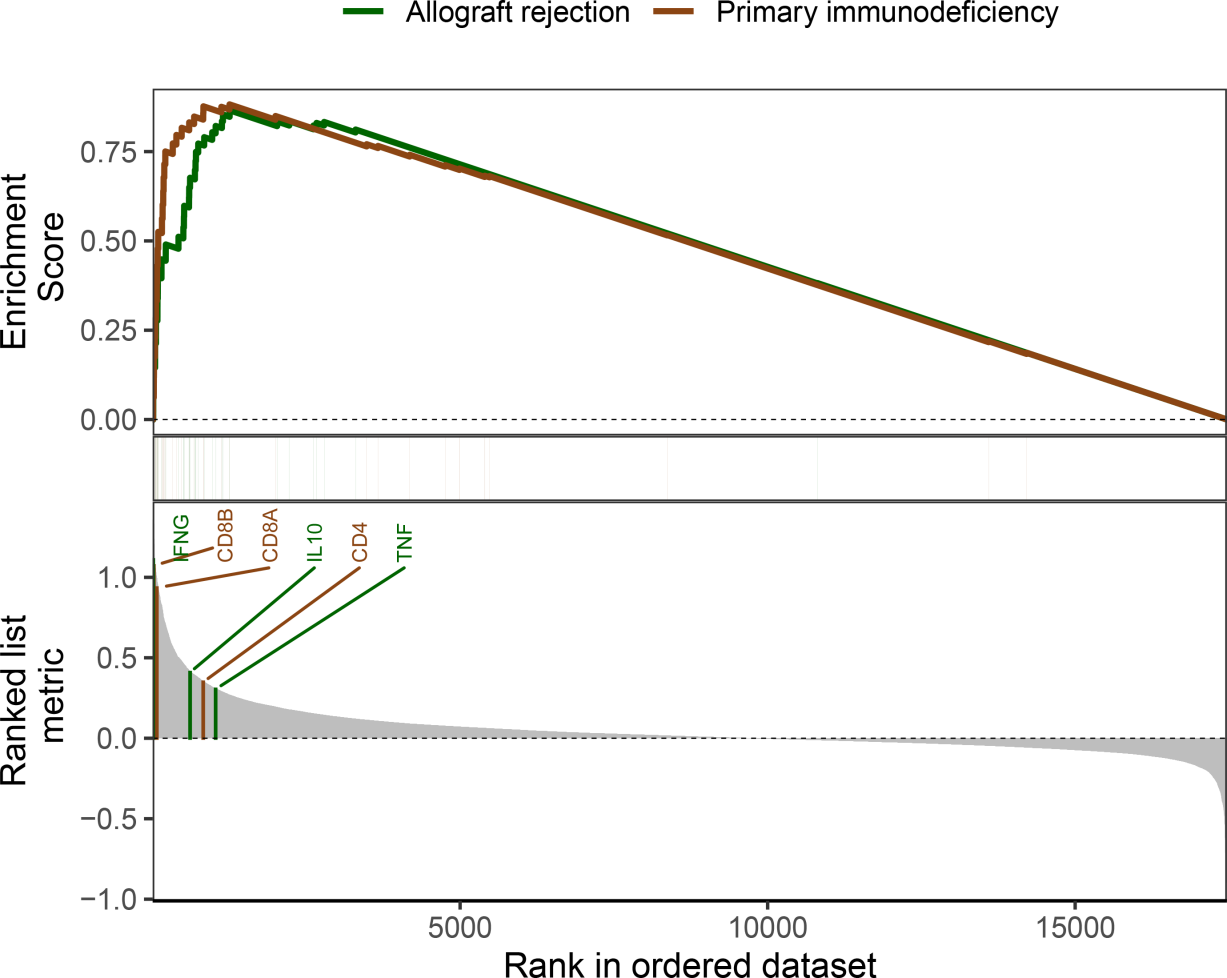
GSEA of signaling pathways between two risk groups.

To further determine salient enriched pathways associated with ICDRGs landscape in BRCA, GSEA was conducted between the high and low-risk groups. Gene sets were dramatically enriched in the immune pathways such as primary immunodeficiency and allograft rejection signaling pathways (**Figure 7**).

### 3.8 Immune characteristics between two risk groups

**Figure 8:**
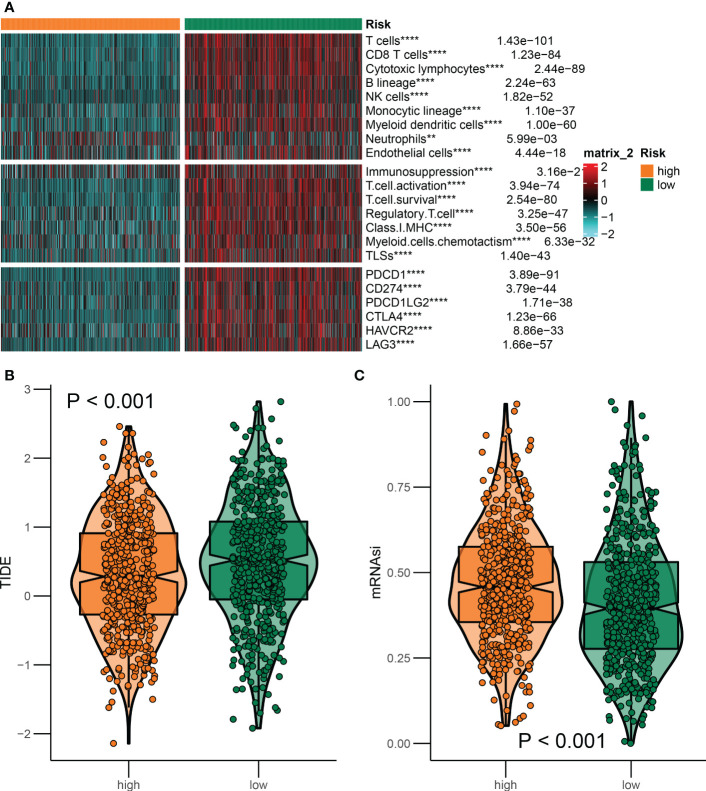
Differences in the immune landscape between the two groups. **(A)** Heatmap of immune characteristics in the high-risk and low-risk groups. **(B, C)** The association of ICDRGs risk score with immunotherapy response. **P<0.01; ***P<0.005; ****P<0.001.

As a general principle, patients with BRCA presented distinct immunological portraits due to different pathological and molecular characteristics (28). It was observed that immune features were markedly distinguished between patients in the high and low-risk groups. As previously mentioned, a restricted panel of chemotherapeutics could provoke a combination of stress and cell death that was immunogenic, thus activating the tumor-specific immune response. Apparently, the immune response including T-cell infiltration and immune checkpoint expression was more robust in the low-risk group compared to the high-risk group (**Figure 8A**). Furthermore, emerging evidence implicated that ICDRGs had an impressive impact on the activation of certain antitumor immune responses. To predict the likelihood of response to immunotherapy, the TIDE algorithm was employed to reveal the tumor immune dysfunction and exclusion score. Incorporating the TIDE algorithm analysis, we discovered that low-risk BRCA patients with lower TIDE scores were more encouraged in reacting to immunotherapy (**Figure 8B**). In addition, patients in the low-risk group had lower cancer stem cell properties, allowing patients to benefit from immunotherapy, thus contributing to improved prognosis (**Figure 8C**).

### 3.9 Correlation between immune subtype and risk subgroups

**Figure 9:**
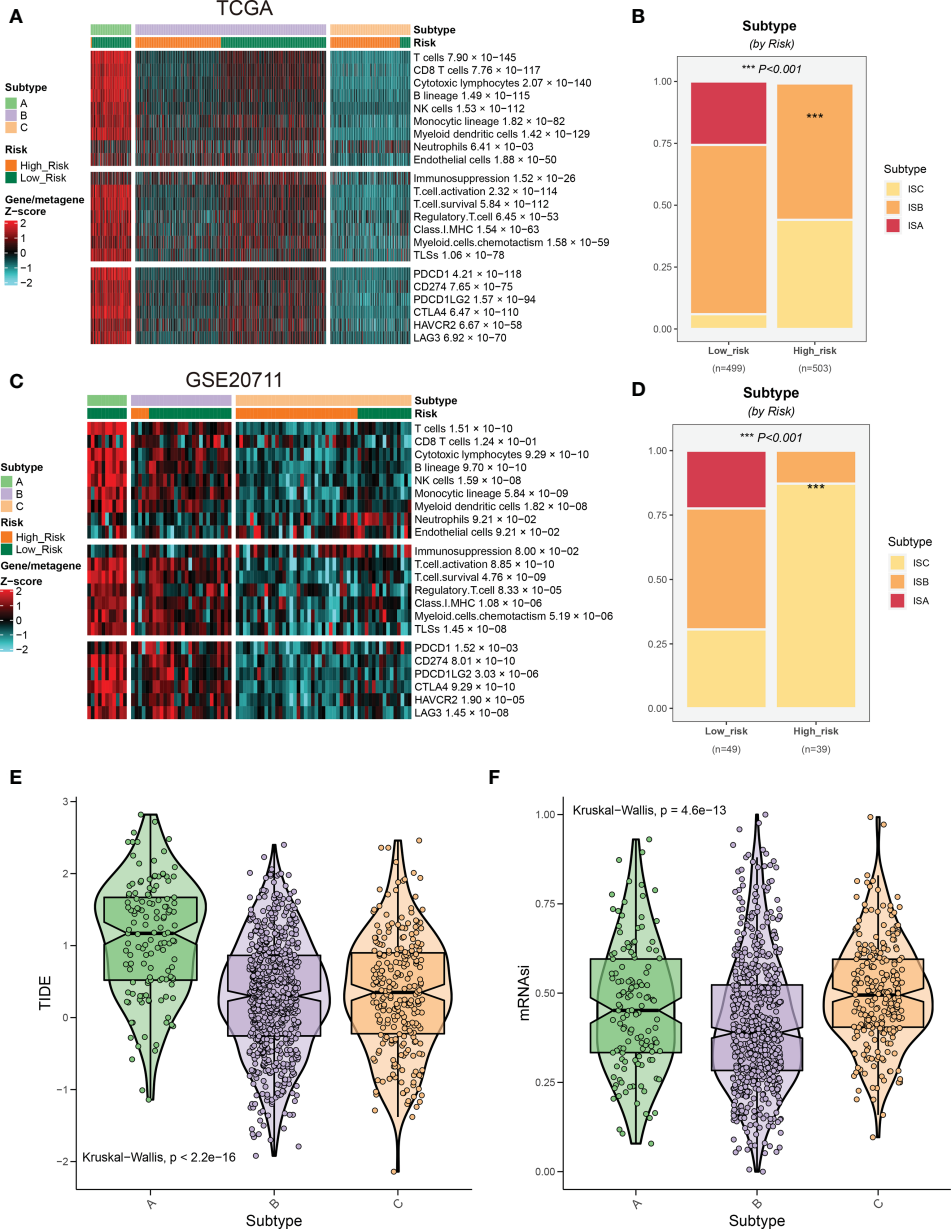
Relationship between risk groups and immune subtypes. **(A)** Patients in the TCGA cohort were divided into three immune subtypes based on the immune infiltrating landscape. **(B)** The distribution of three immune subtypes between the two risk groups. **(C, D)** Validation of the immune subtypes and their distribution in GSE20711. **(E, F)** Association of risk scores of immune subtypes with response to immunotherapy.

In the immune cell infiltration of BRCA tissue, the infiltration of tumor-associated immune cells varies greatly among patients (29). According to the different immune cell infiltrating landscapes, BRCA patients in the TCGA database were divided into three immune subtypes (namely immune subtype A, B, and C; ISA, ISB, and ISC) (**Figure 9A**). Moreover, ISA and ISB predominated in the low-risk group (**Figure 9B**), suggesting patients in the low-risk group exhibited a more vigorous immune response. These findings were also validated in GSE20711 (**Figures 9C, D**). ISA subtypes presented higher TIDE scores and lower tumor stemness, indicating that patients in the ISA subtype were more responsive to immunotherapy (**Figures 9E, F**).

## 4 Discussion

A substantial amount of research and improved clinical processes have accelerated the identification and treatment of BRCA patients, while prognostic and predictive markers are still lacking. More importantly, the exact function of ICDRGs in BRCA development has been increasingly appreciated for their crucial role in the evolution of BRCA as well as their potential as useful fingerprinting biomarkers (21, 30). In the present study, 34 ICDRGs were finally identified to meet our subsequent research. We first conducted the PPI analysis among the 34 ICDRGs in BRCA to reveal their relationships with protein signaling. And the expression of ICDRGs was significantly correlated with prognosis. Among these, overexpressed HAP90AA1 was commonly associated with an unfavorable prognosis, suggesting its potential as a promising prognostic candidate. We also found other crucial ICDRGs, such as IFNG, participated in BRCA progression and had a great impact on the overall survival of patients.

Indeed, IFNG signaling antagonizes both adaptive and innate immune responses through an inhibitory feedback circuit orchestrated by cancer cells. Coerced immune functions could be unleashed through blocking IFNG signaling within tumors, albeit the extent to which each of these effector’s arms contributed to the response largely depended on the immune context (31). Our findings extend the current knowledge by highlighting the essential role of IFNG in BRCA survival. IFNG, a key regulator of immune suppression, may also be a vital determinant in ICD, which can eradicate epibiotic BRCA cells. Thus, targeting IFNG signaling may be an effective approach to ablating the constraint of the immune system and improving therapeutic outcomes.

Patients may have significantly different clinical outcomes despite the exhibition of similar clinicopathological characteristics. As a consequence, patients with early-stage BRCA, in particular, those with ER/PR-positive and HER2-negative cancers, may be overtreated with chemotherapy according to clinical and pathological features alone. Approximately 60% of early-stage BRCA patients endure toxic side effects during adjuvant chemotherapy, of which only a small subset (15%) will finally derive benefit (32). Therefore, there is a growing interest in developing reliable and efficient prognostic biomarkers to precisely recognize patients with high-risk diseases, allowing them to benefit from intensive treatment. Herein, we demonstrated that 6 vital ICDRGs (HSP90AA1, CASP8, PIK3CA, MYD88, CD8A, and CD8B) were identified to be associated with survival by univariate cox and LASSO regression analysis. A risk signature based on the 6 ICDRGs was developed and our risk model had a powerful ability in evaluating prognosis. Subsequently, the BRCA samples were committed into two groups according to the risk score calculated by risk signature. Kaplan-Meir survival curves showed that the high risk subtype was associated with a dismal prognosis. Those conventional characteristics that represent patients’ overall survival mainly comprise tumor size, TNM stage, malignancy grade, and subtype that could be effectively employed for specific subgroups; however, these prognostic profiles have considerable limitations in predicting an individual’s survival outcomes as the presence of tumor heterogeneity (33). Considering this, we integrated the clinicopathological features for a comprehensive prognostic analysis. The results manifested that this risk model had a good performance in predicting the overall survival of BRCA patients and might serve as an independent prognostic indicator in clinical settings.

Due to increased awareness and modern screening methods, the prevalence of BRCA has been increasing over the last decades (34). Traditionally, treatment decisions for BRCA have been driven by risk stratification based on standardized clinicopathologic risk factors. Furthermore, the development of treatment concepts taking into account the heterogeneity of BRCA will make a valuable contribution to the design of individualized treatments (35). In an attempt to find personalized chemotherapy agents for patients with distinct risk groups, we illuminated the sensitivity of Bortezomib, PD.0325901, BMS.536924, AZD6244, Vinblastine, CEP.701, Rapamycin, Roscovitine, PF.02341066, and LFM.A13 between the high-risk and low-risk groups. With the increasing research on molecular targets in tumors, small molecule drugs are receiving increasing attention as a resource for drug discovery (36). With this in mind, we defined several small-molecule drugs for two risk groups. On one hand, some small molecule agents, such as fasudil, TOCK1N.35874, arachidonyltrifluoromethane, MK.886, and X4.5.dianilinophthalimide, displayed a high reactivity to BRCA patients in the high-risk group. On the other hand, drugs like TTNPB, imatinib, NU.1025, PHA.00816795, and AH.6809 seemed to be suitable for patients with low-risk scores.

Increasing emphasis is being placed on the role of TME in BRCA occurrence and development. For example, cancer-associated fibroblasts (CAFs), derived from tissue-resident fibroblasts, frequently confer protection to tumor cells in the context of cytotoxic and targeted therapies (37). Emerging evidence demonstrates that angiogenesis and immunosuppression frequently occur simultaneously in response to this crosstalk between cancer cells and the surrounding microenvironment (38). The anti-angiogenic therapy working in synergy with immunotherapies reshape the TME and improve treatment response (39). Numerous studies have demonstrated various inflammatory immune cells; such as, the high abundance of CD8+ T cells is closely correlated to the immune escape of BRCA (40). Likewise, the landscape of tumor-infiltrating CD8+ and CD4+ T cells is generally associated with the prognosis of BRCA patients (41, 42). Here, we found that robust immune responses including CD8+T cell infiltration and immune checkpoint expression were mainly augmented in the low-risk group, implying the good immunity of the low-risk group. Based on the TIDE scores, we observed that patients in the low-risk group exhibited attenuated stemness. Indeed, recent work demonstrated that fueling Hh signaling in CAFs ascended stemness in BRCA by fibroblast growth factor 5 (25). Tumor stemness was significantly increased in the high-risk group of BRCA patients. Thus, targeting ICDRGs or ICDRGs-associated signaling may be a promising strategy to decrease stemness and improve therapeutic sensitivity.

Recently, the development of immunology has witnessed the prosperity of combining immunogenic therapeutic and new immunotherapeutic regimens for the treatment of malignancies (20, 43). Herein, we identified 3 immune subgroups (ISA, ISB, and ISC) according to the immune infiltrating landscape. This could reflect, to some extent, the fact that patients in distinct immune subgroups exhibited different responses to immunotherapy. Our observations were also confirmed in the external validation set. Transformed tumor cells remodel the TME in their favor, frequently hastening inflammation and pro-tumorigenic microenvironmental communications along with tumor progression of BRCA (44). And such crosstalk can lead to immune signaling dysregulation, which is generally correlated to augmented resistance and cell cytotoxic during targeted therapies (45). Considering that the clinical trial data based on the precision and individualized approaches will make it more convincing and surprising in the ICD therapy, more detailed research will be needed to classify the internal regulation network between ICD signal and immune therapy. Besides, as another limination, experiments on the function of the key ICDRGs in BRCA cell lines and animal models will be further performed, which is also our next research project.

## 5 Conclusion

In conclusion, our study emphasized the association between the ICDRGs and the TME in BRCA. Also, we established an ICDRGs prognostic signature, which proved pronounced value in predicting the survival of BRCA patients. These findings may offer cues to BRCA immunotherapy.

## Data availability statement

The original contributions presented in the study are included in the article/**Supplementary Material**. Further inquiries can be directed to the corresponding authors.

## Author contributions

RZ, MP and TH contributed to the conception and design of the project. RZ, WW, LP, and MP wrote the manuscript. XL, YH, WL, YM and XZ participated in the data collection, analyses and interpretation. RY, SZ and XG reviewed and revised the manuscript. MP and TH guided the process of analysis and writing. All authors contributed to the article and approved the submitted version.
