# Supplementary material for: The prognostic value and response to immunotherapy of immunogenic cell death-associated genes in breast cancer
Source: Front Oncol. 2023 Feb 9;13:1047973. doi: 10.3389/fonc.2023.1047973 (PMC9948621; doi:10.3389/fonc.2023.1047973)
Supplement: Supplementary file 1 [file Table_1.docx]

| Supplementary table 1. Hazard proportionality analysis. | | | |
| --- | --- | --- | --- |
| **Gene** | **chisq** | **df** | **p** |
| NT5E | 7.59E-03 | 1 | 0.931 |
| CALR | 2.56E+00 | 1 | 0.110 |
| HMGB1 | 1.24E-03 | 1 | 0.972 |
| HSP90AA1 | 8.63E-01 | 1 | 0.353 |
| ATG5 | 6.67E-02 | 1 | 0.796 |
| BAX | 2.39E+00 | 1 | 0.122 |
| CASP8 | 3.11E-03 | 1 | 0.956 |
| PDIA3 | 2.35E+00 | 1 | 0.126 |
| EIF2AK3 | 2.78E+00 | 1 | 0.096 |
| PIK3CA | 7.41E-01 | 1 | 0.389 |
| CXCR3 | 8.88E-02 | 1 | 0.766 |
| IFNB1 | 4.49E-01 | 1 | 0.503 |
| IFNA1 | 7.43E-01 | 1 | 0.389 |
| IL10 | 1.35E-01 | 1 | 0.713 |
| IL6 | 1.67E+00 | 1 | 0.197 |
| TNF | 4.80E+00 | 1 | 0.028 |
| CASP1 | 7.25E-01 | 1 | 0.394 |
| IL1R1 | 2.08E-01 | 1 | 0.649 |
| IL1B | 1.44E+00 | 1 | 0.231 |
| NLRP3 | 1.37E-03 | 1 | 0.971 |
| P2RX7 | 1.08E-02 | 1 | 0.917 |
| LY96 | 5.93E-01 | 1 | 0.441 |
| MYD88 | 1.55E+00 | 1 | 0.213 |
| TLR4 | 3.06E-01 | 1 | 0.580 |
| CD4 | 5.80E-01 | 1 | 0.446 |
| CD8A | 1.16E+00 | 1 | 10.281 |
| CD8B | 6.17E-01 | 1 | 0.432 |
| FOXP3 | 2.74E-01 | 1 | 0.601 |
| IFNG | 7.12E-01 | 1 | 0.399 |
| IFNGR1 | 2.19E+00 | 1 | 0.139 |
| IL17A | 1.71E+00 | 1 | 0.191 |
| IL17RA | 1.30E+00 | 1 | 0.255 |
| PRF1 | 5.11E-04 | 1 | 0.982 |
